# Genomics of maize resistance to kernel contamination with fumonisins using a multiparental advanced generation InterCross maize population (MAGIC)

**DOI:** 10.1186/s12870-021-03380-0

**Published:** 2021-12-16

**Authors:** Noemi Gesteiro, Ana Cao, Rogelio Santiago, Rosa Ana Malvar, Ana Butrón

**Affiliations:** 1 Misión Biológica de Galicia (CSIC), Box 28, 36080, Pontevedra, Spain; 2 Departamento Biología Vegetal y Ciencias del Suelo, Facultad de Biología, Universidad de Vigo, Unidad Asociada Agrobiología Ambiental, Calidad de Suelos y Plantas, As Lagoas Marcosende, 36310 Vigo, Spain

**Keywords:** Maize kernel, Fumonisin, Resistance, MAGIC, GWAS

## Abstract

Maize kernel is exposed to several fungal species, most notably *Fusarium verticillioides*, which can contaminate maize kernels with fumonisins. In an effort to increase genetic gains and avoid the laborious tasks of conventional breeding, the use of marker-assisted selection or genomic selection programs was proposed. To this end, in the present study a Genome Wide Association Study (GWAS) was performed on 339 RILs of a Multiparental Advanced Generation InterCross (MAGIC) population that had previously been used to locate Quantitative Trait Locus (QTL) for resistance to Fusarium Ear Rot (FER). Six QTLs for fumonisin content were detected in the bins 3.08, 4.07, 4.10, 7.03-7.04, 9.04-9.05 and 10.04-10.5. Five of the six QTLs collocate in regions where QTLs for FER were also found. However, the genetic variation for fumonisin content in kernel is conditioned by many other QTLs of small effect that could show QTL x environment interaction effects. Although a genomic selection approach to directly reduce fumonisin content in the kernel could be suitable, improving resistance to fumonisin content by genomic selection for FER would be more advisable.

**Supplementary Information:**

The online version contains supplementary material available at 10.1186/s12870-021-03380-0.

## Background

Maize kernel is exposed to several fungal genera, including *Fusarium*, *Aspergillus* or *Penicillium*, which can contaminate maize kernels with mycotoxins. In Spain, *Fusarium verticillioides* predominates over other species. This species produces fumonisins that, in addition to causing multiple disorders in animals [1], have been classified as possibly carcinogenic by the International Agency for Research on Cancer [2].

In order to increase genetic gains and eliminate expensive, labor and time-consuming tasks associated to conventional breeding, such as artificial inoculations and fumonisin quantifications, markers linked to the genes involved in resistance to fumonisin contamination can be used in marker-assisted selection programs. Therefore, many studies have been focused on the detection of Quantitative Trait Locus (QTL) for resistance to Fusarium Ear Rot (FER) [3–8], but fewer locate QTL for resistance to fumonisin contamination [3, 7–9]. FER can be scored on a visual scale, whereas kernel fumonisin quantification is more laborious and expensive, requiring high-performance chromatography or enzyme-linked immunosorbent assays (ELISA). Although screening for FER would be an effective way to improve resistance to fumonisin contamination due to moderate to high correlation coefficients between both traits [10–14], QTL studies for fumonisin content are still necessary because that association has been noted to be weaker in particular genetic backgrounds [13].

Until now, most QTL studies for fumonisin content were done using biparental mapping populations [3, 7, 8]. In recent years, however, mapping approaches involving multiparental designs such as the Nested Association Mapping (NAM) proposed by Yu et al. [15] and Multiparental Advanced Generation InterCross (MAGIC) proposed for crop mapping by Mackay et al. [16] have gained attention. In contrast to inbred panels, multiparental populations largely avoid the potentially confounding influence of population structure and increase the frequency of panel rare alleles which could be of particular interest for breeding [17]. An efficient MAGIC population has clear advantages over other approaches: it has greater genetic variation than biparental populations; it has a balanced allelic frequency since all founders contribute equally; and a uniform and high recombination rate that increases the resolution of genetic analysis, mapping and gene isolation [18].

In the current study, a genome-wide association study (GWAS) approach has been used to find QTL for resistance to fumonisin contamination in an eight-way MAGIC population previously used for locating QTL for FER [10]. In this MAGIC population founders have shown high diversity for specific traits, FER and fumonisin content among those traits [10, 19–25]. In addition, MAGIC populations could be used to obtain superior breeding lines for resistance to FER and to fumonisin contamination [26], or as base materials to perform genomic selection [27]. Thus, the main objectives of this research were: (i) to carry out a GWAS analysis in a MAGIC population in order to identify novel QTLs involved in reduced fumonisin contamination in the maize kernel, and (ii) to propose the most suitable breeding program to reduce kernel contamination with fumonisins.

## Results

**Fig. 1:**
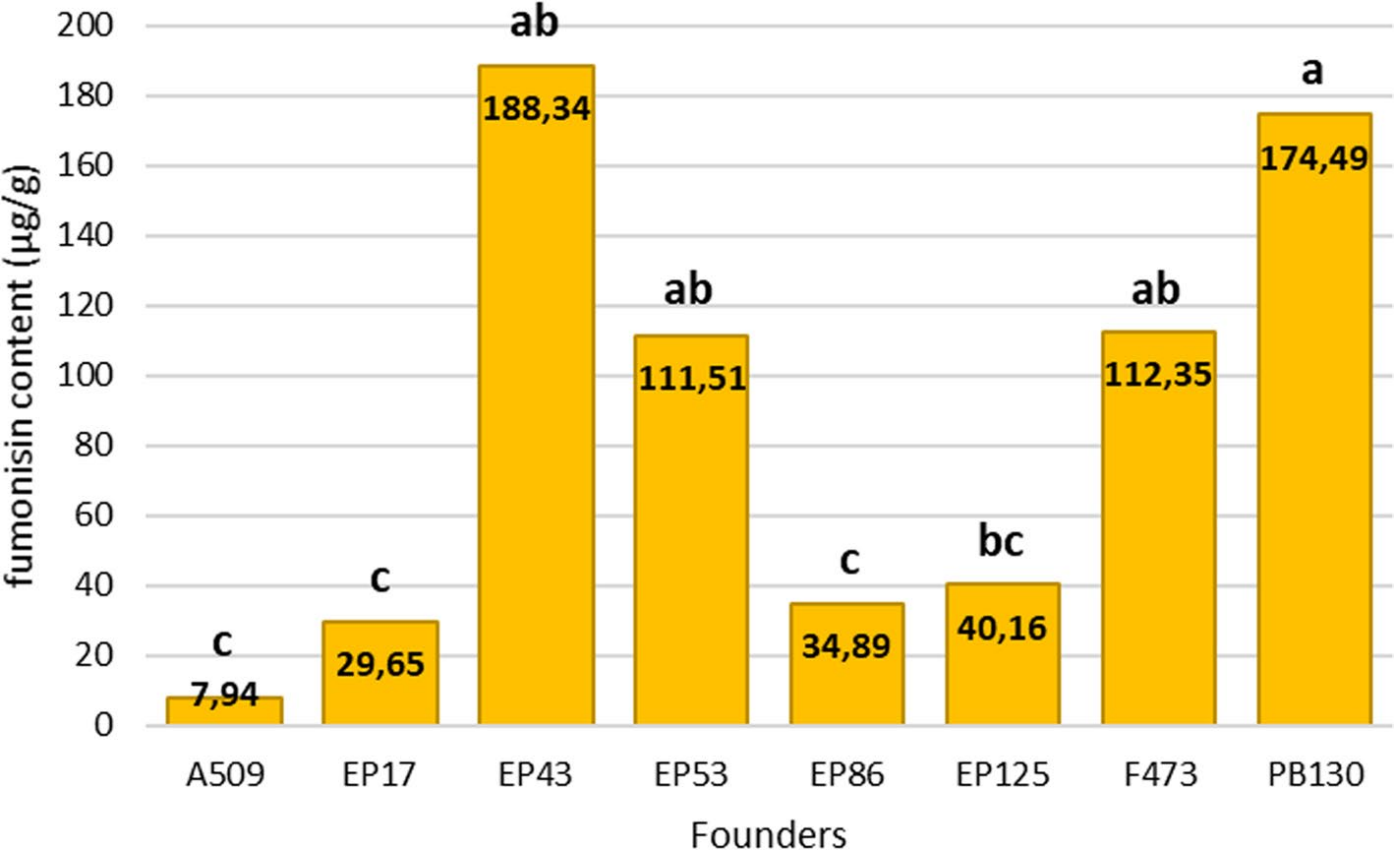
Kernel total fumonisin content in the founders of the MAGIC population of maize in 2014. Different letters indicate significant differences at a significance level of 0.05

Founders were profusely replicated in 2014 trial (16 replicates) meanwhile, in 2016, they were just replicated twice in a trial with 800 plots (rows). Therefore, the estimation of founder mean was considerably less precise in 2016 and no significant differences were found among them in that year. However, significant differences were found among founders for fumonisin content in kernels in 2014 (F = 5.6, *P* > 0.0001) (Fig. 1). Data on fumonisin content of each RIL in 2014 and 2016 are shown in Additional file 1. The genetic heritability for kernel fumonisin content estimated on a mean basis, although low due to significant genotype x environment interaction (Z = 7.28, *p* < 0.0001), was significantly different from zero (0.37 ± 0.067). The phenotypic correlation coefficient between fumonisin content and FER (0.57 ± 0.03) was moderate and significant, while the genotypic correlation coefficient was high (0.92 ± 0.09). Neither significant genotypic (− 0.07 ± 0.11) and phenotypic (− 0.06 ± 0.04) correlation coefficients were found between fumonisin content and days to silking.

**Fig. 2:**
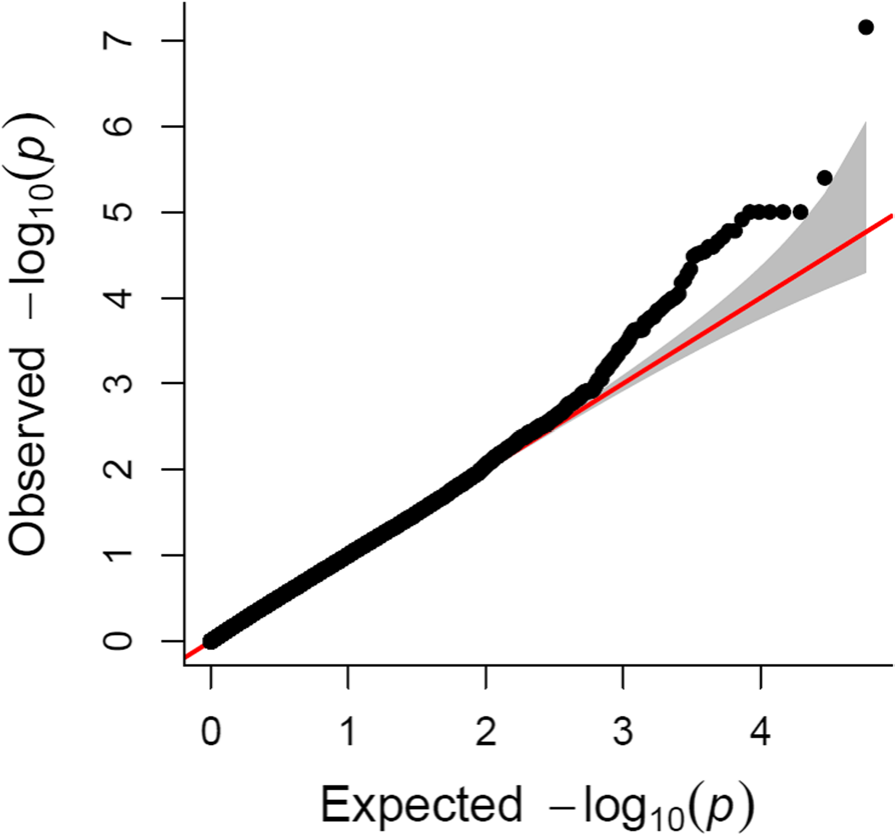
Quantile-quantile plots of a mixed linear model for kernel fumonisin content in the MAGIC population. The dotted lines show the 95% confidence interval for the QQ-plot under the null hypothesis of no association between the SNP and kernel fumonisin content

**Fig. 3:**
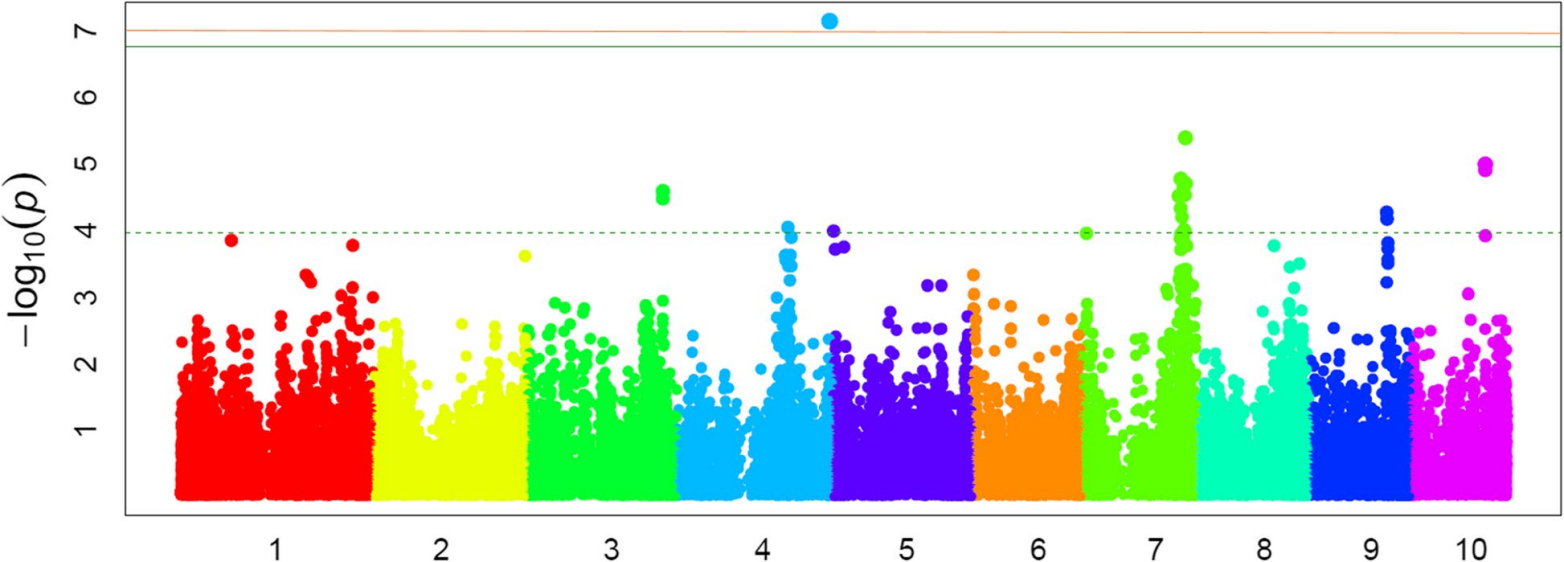
Manhattan plot of a mixed linear model for kernel fumonisin content in the MAGIC population. Single nucleotide polymorphisms (SNPs) above the green horizontal line exceeded the Bonferroni limit established for an experiment-wide error of 0.01 (default threshold supplied by FarmCPU), above the red horizontal line exceeded the alpha = 0.10 genome-wide *p*-value threshold and above the dotted horizontal line the (*p* value = 0.0001) samples fell outside the 95% confidence interval for no significant association between SNP and fumonisin content

Twenty-four SNPs could be considered as putatively linked to QTLs because they were located at the top of the Q-Q plot and presented *p* values < 0.0001 (Fig. 2); those observed *p* values being significantly lower than those expected under no significant association between SNPs and fumonisin content. However, only one of these outliers exceeded the alpha = 0.10 genome-wide *p*-value threshold (empirically estimated as a comparison-wide *p*-value of 7.02E-08; Fig. 3). The approximate support interval for each significant SNP was established by visually checking the linkage disequilibrium (LD) heatmap plot of the genomic region containing the SNP. The support interval was considered to be the region where appreciable LD was observed between SNPs (r^2^ > 0.3). Supporting intervals for QTLs were generally less than 20 Mbp, except for QTLs located at or near centromeres, where LD is exceptionally high.

**Fig. 4:**
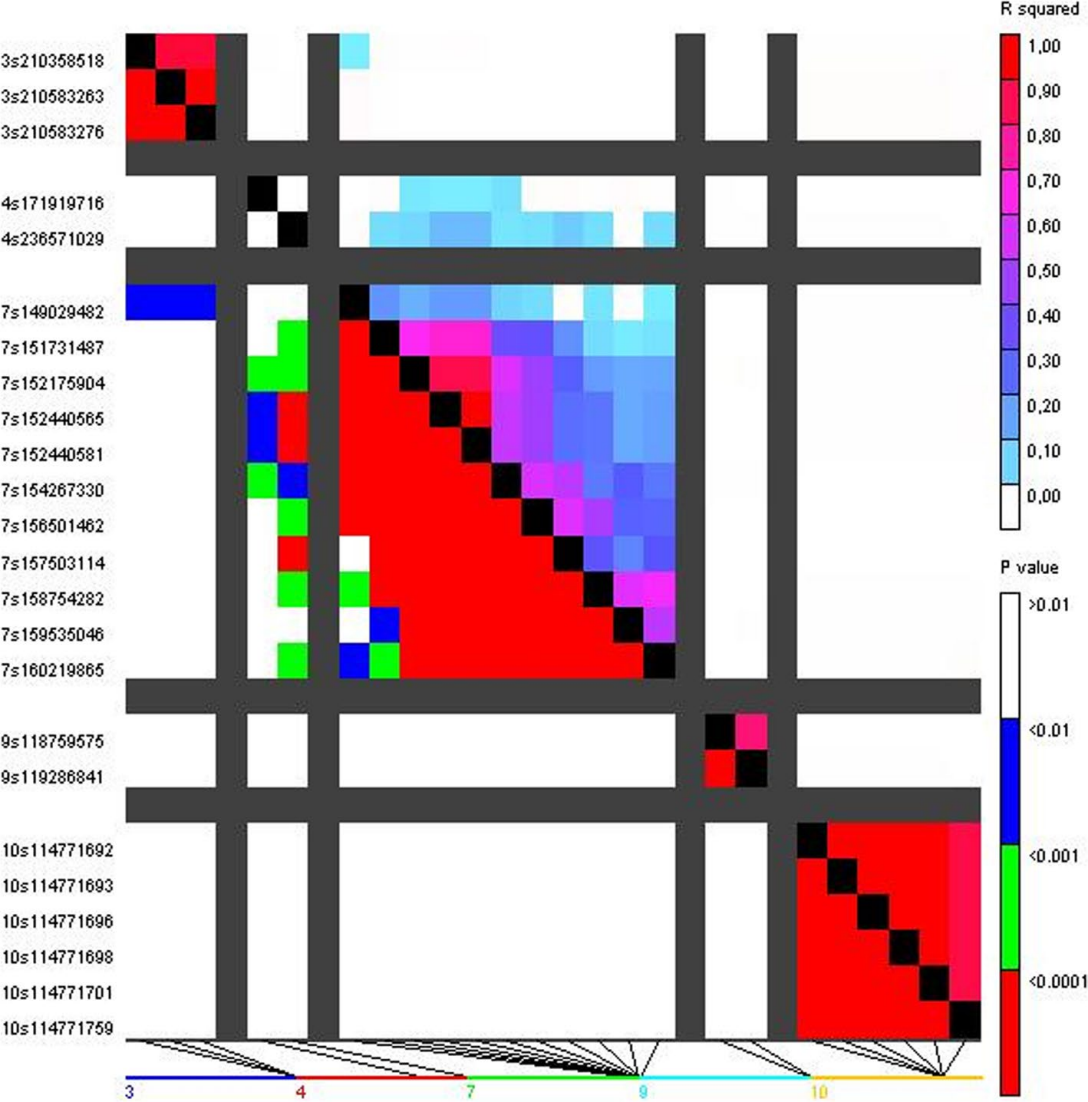
Linkage disequilibrium between SNPs significantly associated with maize kernel fumonisin content

Six putative QTLs were found for fumonisin content at bins 3.08, 4.07, 4.10, 7.03-7.04, 9.04-9.05 and 10.04-10.5 because SNPs with overlapping confidence intervals were clustered in the same QTL. No appreciable LD (r^2^ > 0.2) was found between SNPs located in different QTLs (Fig. 4). The most reliable QTL would be at 235-237 Mb on chromosome 4 (*p* value = 7.0^− 08^) (Table 1). As this region comprises only 2 Mb all genes contained in it were considered as candidate genes but the discussion will focus only in Zm00001d053751 with an annotated function possibly involved in resistance. In addition, it should be noted that, at significant SNPs, the frequencies of unfavorable alleles for kernel fumonisin accumulation were lower than those of favorable alleles.

**Table 1:** QTLs for fumonisin content in kernel found in the MAGIC population in 2 years

Position^a^ (v2)	SNP-containing gene (v4)	SI (v2)^b^	QTL name	QTL bin^c^	R^2d^	Combined *p*	Effect	2014 *p*	Effect	2016 *p*	Effect	Allele proportion^e^
210,358,518	Zm00001d043923	209-217	QTL-3.08	3.08	4.3	3.3E-05	31.3	2.6E-05	48.3	0.002	18.1	39/244
210,583,263	Zm00001d043937					2.6E-05	34.1	4.9E-05	50.1			33/269
210,583,276	Zm00001d043937					2.6E-05	34.1	4.9E-05	50.1			33/269
171,919,716	Zm00001d051945	170-180	QTL-4.07	4.07	4.6	9.0E-05	28.5	0.0054	30.9	0.0001	26.1	47/265
236,571,029	Zm00001d053825	235-237	QTL-4.10	4.10	9.2	7.0E-08	51.1	2.5E-06	68.1	0.0001	34.0	18/273
149,029,482	Zm00001d021505	145-161	QTL-7.03	7.03-7.04	9.7	3.0E-05	35.6	0.001	42.7	0.0003	28.5	27/266
151,731,487	Zm00001d021584					3.1E-05	37.4	0.0001	51.6	0.006	23.1	24/276
152,175,904						4.6E-05	35.1	0.0006	45.3	0.02	25.0	28/277
152,440,565	Zm00001d021599					1.7E-05	37.5	0.0006	45.4	0.0003	29.6	25/267
152,440,581	Zm00001d021599					1.7E-05	37.5	0.0006	45.4	0.0003	29.6	25/267
154,267,330	Zm00001d021668					6.3E-05	35.8	8.3E-05	53.5			24/274
156,501,462	Zm00001d021739					2.2E-05	36.6	4.5E-05	53.5			30/288
157,503,114	Zm00001d021781					9.6E-05	36.8	0.0006	49.0	0.005	24.6	20/277
158,754,282						2.9E-05	36.5	4.5E-05	54.2			29/289
159,535,046	Zm00001d021864					4.0E-06	43.1	1.7E-05	61.3	0.004	24.9	19/270
160,219,865	Zm00001d021902					2.0E-05	37.4	0.0003	47.8	0.0009	27.1	23/259
118,759,575	Zm00001d047169	110-131	QTL-9.04	9.04-9.05	6.4	5.3E-05	39.0	0.0001	56.5	0.0009	25.2	21/290
119,286,841						6.7E-05	36.3	0.0001	53.4			28/300
114,771,692		95-132	QTL-10.04	10.04-10.05	5.2	1.0E-05	38.4	3.5E-06	61.2	0.0004	36.2	29/287
114,771,693						1.0E-05	38.4	3.5E-06	61.2			29/287
114,771,696						1.0E-05	38.4	3.5E-06	61.2			29/287
114,771,698						1.0E-05	38.4	3.5E-06	61.2			29/287
114,771,701						1.00E-05	38.4	3.5E-06	61.2			29/287
114,771,759						1.22E-05	38.9	9.4E-06	59.8			29/287

^a^ Base pair position of the significant SNP

^b^ Supporting interval (SI) of the QTL, region in which appreciable linkage disequilibrium (LD) is observed between single-nucleotide polymorphisms (SPNs) (r^2^ > 0.3)

^c^ Bin where QTLs are located

^d^ R^2^: proportion of phenotypic variability explained by the QTL in the combined analysis; *p*: *p*-value for the association between polymorphic variation at the SNP and phenotypic variation for fumonisin content in combined and individual analyses; Effect: difference between the average phenotypic values of the homozygotes for the less and most frequent alleles in combined and individual analyses

^e^ Number of RILs with favorable/unfavorable alleles for each significant SNP

## Discussion

Large and significant differences among inbred founders were detected for fumonisin content, but the estimated heritability for fumonisin content in the MAGIC population derived from those founders was low highlighting the importance of moving to breeding methodologies assisted by molecular markers with stable effects across environments. Appreciable additive genetic variability among the RILs of the MAGIC population was found because the heritability estimate, although low, was significantly different from zero and similar to those obtained for other genetically diverse populations [7, 9, 12, 14, 28]. These results also confirmed that the kernel inoculation technique would be suitable to detect differences for fumonisin content among genotypes, probably because would guarantee a more homogeneous dispersal of the inoculum across genotypes than other inoculation techniques [8, 29, 30].

In the current MAGIC population, genetic variation for kernel fumonisin content was not associated to variability for days to silking as it often occurred among other materials [3, 7, 9] Therefore, breeding for resistance to fumonisin content in this MAGIC population would not result in unintended maturity changes that would compromise adaptation to specific environments, an important advantage over other mapping populations. The smaller linkage blocks and reduced population structure of this MAGIC population compared to bi-parental populations and association panels, respectively, could allow independent segregation of both traits [10].

According to other studies, kernel fumonisin content was highly correlated with FER and, consequently, colocalizations between QTLs for FER and fumonisin content were expected [9–12, 14]. Five out of the six QTLs found for fumonisin content were located in genomic regions where QTLs for FER were found in a previous study using the same population [10]. However, there are studies in which high concentrations of fumonisins were found in visually FER resistant genotypes and, in those cases, the QTL conferring resistance/susceptibility to fumonisin contamination would not show any effect on FER [12, 13, 31]. According to these observations, the most reliable QTL for fumonisin content was found in a region where no QTL for FER were previously detected [10].

Among genes contained in the supporting interval of the QTL at bin 4.10, a gene annotated as *negative regulator of systemic acquired resistance* (SNI1), Zm00001d053751, is proposed as a good candidate gene for that QTL. In *Arabidopsis*, disruption of SNI1 function resulted in enhanced sensitivity to salicylic acid and increased resistance response to pathogens; SNI1 being considered as a subunit of the SMC5/6 complex that plays critical roles in DNA damage responses including DNA damage repair [32, 33]. In addition, Wang et al. [34] showed that SNI1 is a negative regulator of E2F transcriptor factors and could play dual roles in DNA damage responses by linking cell cycle checkpoint and DNA repair. However, recent investigations have showed that SNI1 is not directly involved in systemic acquired resistance or DNA damage accumulation*,* as it was previously proposed, but have suggested that the gene *sni1* could be involved in regulating or signaling immunity [35, 36]. Sanchez-Rangel et al. [37] suggested that activation of the salicylic acid pathway will induce cell death and could facilitate necrotroph proliferation of *Fusarium verticillioides*. Therefore, as SNI1 is involved in salicylic acid-mediated response, we hypothesize that the *sni1* gene could have effect on fumonisin content without affecting FER because it could be involved in conditioning the change from biotrophic to necrotrophic behavior of the fungus.

Many small-effect QTLs appear to be involved in maize resistance to fumonisin contamination, so the value of targeting the significant SNPs to improve resistance to fumonisin contamination of grain is limited because each SNP explains a small percentage of variation [7, 38, 39]. In accordance with that, marker assisted selection based on the QTLs found in the current study, although can serve to discard genotypes extremely contaminated, could not support the pyramiding of minor favorable alleles whose effects were probably hidden by residual and QTL x environment variation. However, genes behind the QTL detected could deserve to be positionally cloned as they seemed to have important effects on fumonisin content across populations [3, 7, 9]. Although the search of QTLs directly involved in resistance to fumonisin contamination uncovered a QTL which was not found when looking for QTLs for FER, lowest stability of genotypes across environments for fumonisin content was observed compared to FER (family- based heritabilities for FER and fumonisin content were 0.56 and 0.37, respectively) [10]. The plot estimate for FER was the average of five individual (ears) values [10], whereas fumonisin content was determined in a sample of the flour obtained from those ears and distribution of mycotoxins in maize flour is not totally homogeneous [40, 41]. Therefore, the data collection itself may contribute to those differences between heritability estimates because lower accuracy for fumonisin content than for FER is expected.

Marker assisted selection based on the QTLs found in the current study could not support the pyramiding of minor favorable alleles whose effects were probably hidden by residual and QTL x environment variation. Considering that the heritability of fumonisin content is low and that each significant SNP explains a small proportion of the phenotypic variability, a genomic selection program to directly reduce fumonisin content would be appropriate, but indirect selection through genomic selection for FER would be more advisable because genetic correlation between both traits is very high and FER evaluation is more cost efficient.

## Conclusions

In conclusion, the current MAGIC population allows finding QTLs involved in resistance to fumonisin accumulation in maize kernel and, its population structure allows independent segregation of genes favouring resistance enhancement without compromising other aspects. However, genetic variation for fumonisin content in maize kernel is conditioned by genotype x environment interaction and many small effect QTLs. Although a direct genomic selection approach to reduce fumonisin content could be suitable, improving resistance to fumonisin accumulation by genomic selection for FER could be easier and more cost efficient.

## Methods

A MAGIC population of recombinant inbred lines (RIL) was developed from the synthetic variety EPS21 as described previously ([10, 20]. EPS21 synthetic was composed of eight unrelated inbred lines, which had in common the lack of “Reid” materials in their pedigrees. The inbred lines used to construct the EPS21 synthetic were A509, EP17, EP43, EP53, EP86, EP125, F473 and PB130 that showed contrasting levels of resistance to FER and fumonisin accumulation in kernel in preliminary evaluations [21]. Inbreds EP17, EP86, EP125 and A509 were partially resistant to fumonisin accumulation, while EP43, EP53, F473 and PB130 were susceptible. The set of RILs from the MAGIC population together with the eight parental founders included as testers were evaluated for resistance to kernel fumonisin accumulation in Pontevedra, Spain (42 ° 24 ′ N, 8 ° 38 ′ W, 20 m above sea level) in 2014 and 2016. In 2014, shortage of seed amount for the MAGIC RILs made us to opt for an experimental design in which those materials were unreplicated, but checks (parental founders of the MAGIC) were profusely replicated to account for block effects. As seed amount for each RIL was increased in 2015, all entries could be replicated in the experimental design used in 2016. In 2014, 672 RILs and the eight founders were evaluated using an augmented design with 16 blocks. The 672 RILs were un-replicated and randomly assigned to 16 blocks. The eight inbred founders were replicated and randomly assigned to plots within each of the 16 blocks, i.e. each block consisted of 42 non-replicated RILs plus the eight founders. The experimental plot consisted of a 13-plant row with a spacing of 0.18 m between plants and 0.8 m between rows. In 2016, 695 RILs and seven parental founders (no seed from PB130 was available) were evaluated using a 26 × 27 α-lattice design with two replications. Each experimental plot consisted of one 19-plant row with a spacing of 0.18 m between plants and 0.8 m between rows.

In both years, five primary ears were inoculated in each plot using a kernel inoculation technique with a four-needle vaccinator: approximately 7-14 days after silking (when half of the plot plants showed visible pistils or silks), the main ears of five plants were inoculated with 2 ml of a spore suspension (10^6^ spores/ml) of an isolate of *F. verticillioides* [42]. Harvesting of each plot was made two months after inoculation. Collected ears were dried at 35 °C for one week, shelled and a representative kernel sample of approximately 60 g was ground using a 0.75 mm sieve in a Pulverisette 14 rotor mill (Fritsch GmbH, Oberstein, Germany). The ground samples were sent to the service “Microbiological Quality in the Agro-Food Sector” of the University of Lleida, Spain, for the determination of total fumonisins (fumoninins B_1_, B_2_ and B_3_) using a commercial ELISA kit (R-Biopharm Rhône Ltd., Glasgow, Scotland, United Kingdom). The recovery rate of the test was approximately 60% with an average coefficient of variation of approximately 8%; specificities for B_1_ were 100%, for B_2_ approximately 40% and for B_3_ close to 100%. The detection limit was 0.025 ppm (mg kg^- 1^). Both sample extraction and preparation, as well as test performance, were carried out as described in the commercial kits.

The GWAS was performed with 339 RILs that presented complete phenotypic data in both years and heterozygosity levels below the 15%. The RILs were previously genotyped for approximately 1000,000 SNPs using a genotyping-by-sequencing (GBS) strategy at the Cornell University Biotechnology Institute. Monomeric and multiallelic SNPs and insertion/deletion polymorphisms were excluded as well as SNPs with more than 20% of missing data or with allele frequency (MAF) less than 5%. A total of 58,556 filtered SNPs distributed across the maize genome were used for GWAS.

First, a combined analysis of variance for kernel fumonisin content was performed using the PROC MIXED procedure of SAS software with RILs as fixed effects and years, replications, years x RILs and blocks as random effects. The best linear unbiased estimator (BLUE) was calculated for each RIL in each year and average RIL data across years were used to perform GWAS analysis.

Means for fumonisin content of inbred founders, calculated by the least square procedure, were compared by Fisher’s protected least significant difference (LSD) and only data recorded in 2014 were analyzed because inbred founders were feebly replicated in the trial of 2016. The heritability (h^2^) for kernel fumonisin content in the MAGIC population was estimated on a RIL-mean basis [43]. Genotypic (r_g_) and phenotypic (r_f_) correlations between kernel fumonisin content and previously published data [10] such as FER and days to silking (these data come from the same trials) were calculated following Holland [44].

GWAS analysis based on a linear mixed model was performed using FarmCPU [45]. We calculated an empirical comparison *p*-value threshold using 1000 data permutation runs and assuming a wide-experiment error of 0.10 (7.02E-08). As, in the Q-Q plot (Fig. 2), F test statistics significantly deviated from the expected F test statistics at *p* < 0.0001, SNPs with *p* values lower than 0.0001, although less reliable than those below the empirical threshold, deserve some consideration. The GWAS analysis was completed with an analysis of linkage disequilibrium (LD) between SNPs significantly associated with kernel fumonisin content using the version 5 of TASSEL [46]. A support interval for significant association was established, and all genes contained in support intervals smaller than 2 Mb were considered as candidate genes and identified and characterized using MaizeGBD genome browser [47]. The search for candidate genes was performed on version 4 of the B73 sequence (Zm-B73-REFERENCE-GRAMENE-4.0).

## Supplementary Information

**Additional file 1.** Average fumonisin content (μg/g) of each MAGIC RIL in trials performed in 2014 and 2016.

## Data Availability

The datasets used and/or analysed during the current study are available from the corresponding author on reasonable request.
